# Nonoxid-HMGB1 Attenuates Cognitive Impairment After Traumatic Brain Injury in Rats

**DOI:** 10.3389/fmed.2022.827585

**Published:** 2022-04-11

**Authors:** Jun-Quan Chen, Shuang-Qi Gao, Lun Luo, Zong-Yuan Jiang, Chao-Feng Liang, Hai-Yong He, Ying Guo

**Affiliations:** Department of Neurosurgery, The Third Affiliated Hospital, Sun Yat-sen University, Guangzhou, China

**Keywords:** cognitive impairment, redox state, HMGB1, traumatic brain injury, SH3RF2

## Abstract

Traumatic brain injury (TBI) is a major global burden of health. As an accepted inflammatory mediator, high mobility group box 1 (HMGB1) is found to be effective in facilitating neurogenesis and axonal regeneration. SH3RF2 (also known as POSHER), an E3 ligase SH3 domain-containing ring finger 2, belongs to the SH3RF family of proteins. Here, we aimed to investigate the role of redox states of HMGB1 on neurite outgrowth and regeneration both *in vitro and in vivo*. In this study, distinct recombinant HMGB1 redox isoforms were used. Sequencing for RNA-seq and data analysis were performed to find the potential downstream target of nonoxid-HMGB1 (3S-HMGB1). Protein changes and distribution of SH3RF2 were evaluated by western blot assays and immunofluorescence. Lentivirus and adeno-associated virus were used to regulate the expression of genes. Nonoxid-HMGB1-enriched exosomes were constructed and used to treat TBI rats. Neurological function was evaluated by OF test and NOR test. Results demonstrated that nonoxid-HMGB1 and fr-HMGB1, but not ds-HMGB1, promoted neurite outgrowth and axon elongation. RNA-seq and western blot assay indicated a significant increase of SH3RF2 in neurons after treated with nonoxid-HMGB1 or fr-HMGB1. Notably, the beneficial effects of nonoxid-HMGB1 were attenuated by downregulation of SH3RF2. Furthermore, nonoxid-HMGB1 ameliorated cognitive impairment in rats post-TBI *via* SH3RF2. Altogether, our experimental results suggest that one of the promoting neurite outgrowth and regeneration mechanisms of nonoxid-HMGB1 is mediated through the upregulated expression of SH3RF2. Nonoxid-HMGB1 is an attractive therapeutic candidate for the treatment of TBI.

## Introduction

Traumatic brain injury (TBI) is a major cause of death and disability around the world (1, 2). Survivors often suffer from a variety of neurological symptoms, such as cognitive dysfunction, disorders of balance, paresthesia, memory problems, etc. (3, 4). The mechanism of brain damage after TBI has been proved to involve both direct mechanical damage and indirect damage (5, 6). Direct mechanical damage results from initial impact and is considered irreversible. Secondary damage is mainly caused by the delayed neurochemical process such as excitotoxicity, mitochondrial dysfunction, oxidative stress, and inflammation, which is reversible (7). However, despite extensive research into the process of TBI disease, there is, still, a lack of effective treatments to promote neurological recovery, and the prognosis remains unfavorable.

HMGB1 (high mobility group box 1) is a nuclear protein, but extracellular HMGB1 works as a damage-associated molecular pattern (DAMP) to stimulate the innate immune system (8, 9). HMGB1 exerts varying biological activities according to the redox states of cysteines, which are at positions C23, C45, and C106 within a protein (10–12). Three isoforms of HMGB1 have been identified, namely, fully reduced HMGB1 (fr-HMGB1), disulfide HMGB1 (ds-HMGB1), and fully oxidized HMGB1 (ox-HMGB1) (13). They interact with different pathogen recognition receptors to participate in different pathophysiological processes. Fr-HMGB1 exhibits that the three cysteines are in the thiol state (reducing status). It elicits intracellular actions *via* binding to RAGE and/or CXCR4 (14). In contrast, in ds-HMGB1, a disulfide bond is formed between C23 and C45 residues due to the oxidation, while C106 remains unchanged. It induces cytokine production *via* binding to TLR4 (15). The final variant, namely, ox-HMGB1, is reportedly non-active, with all three cysteines terminally oxidized (16).

In TBI, HMGB1 has been shown to enhance neuroinflammation and subsequently exacerbate neurocognitive impairment (17, 18). Extracellular HMGB1 is released by necrotic neurons and other immune cells recruited to the injury site (19). It can act both as a chemoattractant for leukocytes and as a proinflammatory mediator to induce the release of proinflammatory cytokines (12). However, the critical role of HMGB1 in facilitating neurogenesis and neural regeneration is neglected. It has been reported that HMGB1 stimulates hippocampal and cortical neurogenesis post-TBI (20). HMGB1 is upregulated in axons of injury-conditioned neurons and enhances axon outgrowth (21). Furthermore, a previous study shows that the overexpression of HMGB1 in motoneurons promotes neuroregeneration in SCI (spinal cord injury) (22). Together, these observations suggest that HMGB1 plays dual and antagonistic roles during neurogenesis and neuroregeneration after CNS (central nervous system) injury.

In this study, to investigate the effects of HMGB1 redox isoforms on neurite outgrowth and regeneration *in vitro*, fr-HMGB1, ds-HMGB1, and nonoxid-HMGB1 (3S-HMGB1) were selected. Nonoxid-HMGB1 is a mutant to mimic fr-HMGB1 functions in which all cysteines are replaced with serines and maintains structural stability in oxidizing milieu (23). In addition, we assessed the effects of nonoxid-HMGB1 in TBI rats and tried to explore the potential molecular mechanism.

## Materials and Methods

### Animals

The post-natal day 1–3 Sprague-Dawley (SD) rats were provided by Southern Medical University SPF Animal Experimental Center (Guangzhou, China). Male adult SD rats were purchased from the Vital River Laboratory Animal Technology Co., Ltd. (Beijing, China), weighing 190–210 g (8–10 weeks old). The animals were fed in a standard environment with a light-dark cycle (12/12-h day/night, 25°C) and had 1 week to adapt to the new environment before surgery. All the animal studies were approved by the Review Committee for the Use of Human or Animal Subjects of Sun Yat-Sen University.

### Primary Culture of Cortical Neurons

Cortical neurons were isolated from the neonatal rats under a microscope. In brief, cerebral cortices were isolated carefully. The separated cortical tissue was shredded and dissociated with a papaya enzyme (Sigma-Aldrich, USA). In the first 2 h, cells were cultured in a high-glucose DMEM-F12 (Gibco, USA) medium. After that, the medium was changed into a neurobasal medium (Gibco, USA). The neurons were cultured in a 5% CO2 incubator for 7 days, and then treated with three different forms of recombinant HMGB1 (ds-HMGB1, fr-HMGB1, and nonoxid-HMGB1; 100 ng/ml, respectively; HMGBiotech, Italy). The dosage of HMGB1 referred to a previous research (24).

### Immunofluorescence

Cortical neurons were fixed with paraformaldehyde for 15 min, and then permeabilized with 0.3% Triton X-100 for 30 min. After washing with PBS, the cells were blocked with 5% BSA for 1 h at room temperature, followed by incubation with a MAP2 antibody (1:100; Abcam, UK), a NeuN antibody (1:100; Millipore, USA), and an SH3RF2 antibody (1:100; Novus, USA) overnight at 4°C. The day after, neurons were incubated with Alexa Fluor 488-conjugated (1:500; Abcam, UK) and Alexa Fluor 555-conjugated (1:500; Abcam, UK) for 1 h at room temperature. Finally, nuclei were visualized with DAPI (1:1,000; Abcam, UK). Images were acquired with a confocal laser scanning microscope (LSM 780; Zeiss, Germany) and analyzed by Image J software.

### Bioinformatics Analyses

Total RNA was isolated from cultured cortical neurons. Sequencing for RNA-seq and data analysis were performed at JinWeiZhi (Suzhou) BIOTECHNOLOGY LLC (https://www.genewiz.com.cn/). In the present analysis, an FDR below 0.05 was identified as the criterion for differentially expressed genes (DEGs). Differences in the mRNA expressions were displayed on heatmaps. GO pathway enrichment analyses were performed to find possible biological processes and signaling pathways associated with the correlated target genes of neurite outgrowth and regeneration.

### Western Blotting Analysis

As previously described (25), 30 μg protein samples were separated by 10% SDS-PAGE gel, and then transferred to polyvinylidene fluoride (PVDF, pore size, 0.45 um) membranes (Millipore Billerica, USA). The membranes were blocked with 5% non-fat dry milk for 1 h, followed by incubation overnight at 4°C with the following primary antibodies: rabbit anti-SH3RF2 (1:1,000), rabbit anti-HMGB1 (1:1,000), mice anti-EGFP (1:1,000, mice anti-GAPDH (1:1,000), and mice anti-β-actin (1:1,000). The day after, the membranes were incubated with secondary antibody HRP-conjugated goat anti-rabbit (1:3,000) or an HRP-conjugated goat anti-mouse (1:3,000) at room temperature for 1 h. The secondary antibody was diluted with 1% non-fat dry milk. Specific bands were detected with a GE AI600 system. ImageJ software was used to quantify the expression of protein.

### Lentiviral Transfection

The lentivirus targeting SH3RF2, pLKD-CMV-mcherry-2A-puro-U6-shSH3RF2, was purchased from Obio Technology Corp., Ltd (Shanghai, China). pLKD-CMV-mcherry-2A-puro-U6 served as negative control. Multiplicity of infection (MOI) was 20. Neurons were transfected at day in Culture 7, and the effect of gene interference was verified after 72 h using western blot assays.

### Stereotaxic Injection

Gene overexpression *in vivo* was achieved by ScAAV vectors. ScAAV vectors carrying Nonoxid-HMGB1 (3S-HMGB1), ScAAV-hSyn-HMGB1 (C23S C45S C106S)-EGFP-WPREs, were purchased from BrainVTA Co., Ltd (Wuhan, China). ScAAV- hSyn-EGFP-WPREs served as negative control. After being anesthetized, rats were placed in a stereotaxic apparatus (RWD, CN). The hole was drilled above the right hippocampus, and viruses were microinfused *via* a 10-μl Hamilton microsyringe. The stereotactic coordinates were as follows: bregma −3. mm, midline 3. mm, 2.5 mm below dura.

### Establishment of the TBI Rat Model

The controlled cortex injury (CCI) device (RWD68099II, China) was used to establish the TBI model. A diameter craniotomy of 5. mm on the right parietal (centered 3. mm posterior and 3. mm lateral from the Bregma) was performed to expose the dura. The CCI was delivered to a depth of 1. mm at a velocity of 5 m/s with a duration of 500 ms. Sham rats received the same craniotomy but not brain injury.

### Isolation, Culture, and Transfection of BMSCs

As previously described, BMSCs were harvested from the femurs and tibias of neonatal rat femurs (26). The adherent cells were passaged when they reached 80~90% confluency, and P3 BMSCs were used for subsequent experiments. The nonoxid-HMGB overexpression plasmids carrying EGFP were purchased from BrainVTA Co., Ltd (Wuhan, China). All plasmids were transfected into BMSCs using Lipofectamine 2000 (Invitrogen, USA).

### Isolation and Identification of BMSC-Derived Exosomes

When BMSCs reached 70~80% confluence, a medium was replaced. After 48 h, the medium was collected. The exosomes were extracted through traditional ultracentrifugation and preserved in a freezer at −80°C. The morphology of exosomes was observed under a transmission electron microscope (FEI, CZ). A BCA protein quantification kit (Beyotime, China) was used to detect protein content. Western blot was used to identify the expression of Exo-specific markers TSG101 (Abcam, USA) and Flotillin-1 (Abcam, USA). Exosomes (5 ug) were injected into the caudal vein after TBI (27).

### Behavioral Procedures

Before testing, rats were transferred to the testing room and adapted to the surroundings for at least 1 h.

#### Open Field Test (OF)

OF test was used to measure locomotor activity of rats post-TBI. The rats were individually placed in an open-field chamber (120 cm × 120 cm × 40 cm) and allowed to explore for 5 min freely. The total distance traveled was used to evaluate motor function, which was recorded by SMART 3.0 software.

#### Novel Object Recognition Test (NOR)

NOR test is widely used to evaluate object recognition memory (28). The discrimination of recognition novelty was assessed by preference index (PI) (time exploring the new object)/(total time spent exploring both objects) (29).

### Statistical Analysis

In this study, data were expressed as mean ± SEM and analyzed by employing the GraphPad 8.0 software (San Diego, USA). The differences between any given two groups throughout this study were analyzed by unpaired Student's *T*-tests, unless otherwise specified. In different treatment groups, one-way ANOVA followed by Fisher's LSD test was employed. Multiple comparisons involving more than one variable were analyzed by two-way ANOVA followed by Tukey's *post-hoc* test. Two-tailed *p* < 0.05 was considered as statistically significant.

## Results

### Nonoxid-HMGB1 and fr-HMGB1 Promote Growth of Primary Cortical Neurons

Extracellular HMGB1 has been evaluated to be a DAMP. Interestingly, HMGB1 has also been found to promote neurite outgrowth and regeneration. Different redox isoforms of HMGB1 may play different roles. We first characterized the cultured cortical neurons with two specific markers (MAP2 and NeuN), and the cells were confirmed as neurons (Supplementary Figure 1).

**Figure 1:**
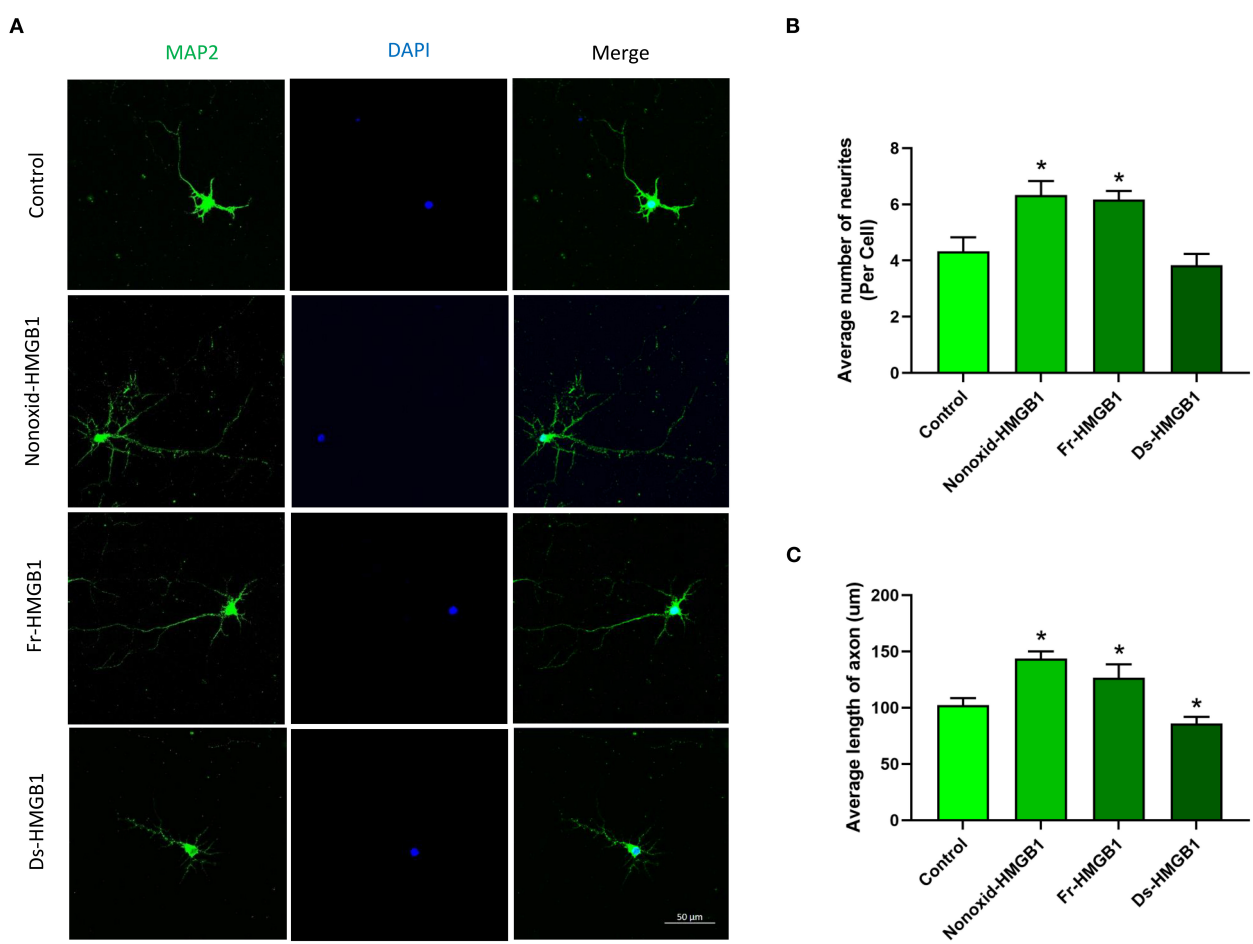
Effects of HMGB1 in different redox forms on neurite outgrowth and axon elongation of cortical neurons. **(A)** Immunocytochemistry was used to label neurites and axons after 24 h of treatment with HMGB1. **(B)** Nonoxid-HMGB1 and fr-HMGB1 significantly increased the average number of neurites compared to the control group. Ds-HMGB1 failed to significantly affect neurite outgrowth. **(C)** Nonoxid-HMGB1 and fr-HMGB1 significantly increased the average axon length compared to control; however, the average axon length was decreased after ds-HMGB1 treatment. Mean + SEM. *p < 0.05.

The biological activity of extracellular HMGB1 is determined by the redox state (30). We tested the role of the nonoxid-HMGB1 and fr-HMGB1 as well as ds-HMGB1 in neuron growth. Compare with the control group, a significantly increased number of neurites were detected in the nonoxid-HMGB1 group and the fr-HMGB1 group (Figures 1A,B). Furthermore, average axon length was measured in the nonoxid-HMGB1 group and the fr-HMGB1 group, and both were significantly increased than the control group (Figure 1C). The average number of neurites was not affected by ds-HMGB1 (Figure 1B). However, the axon length was decreased after ds-HMGB1 treatment (Figure 1C). Thus, these results confirmed that HMGB1-mediated neurite outgrowth and axon elongation of neurons require the reduced state of the protein.

### Differential Gene Expression and Functional Enrichment Analysis

**Figure 2:**
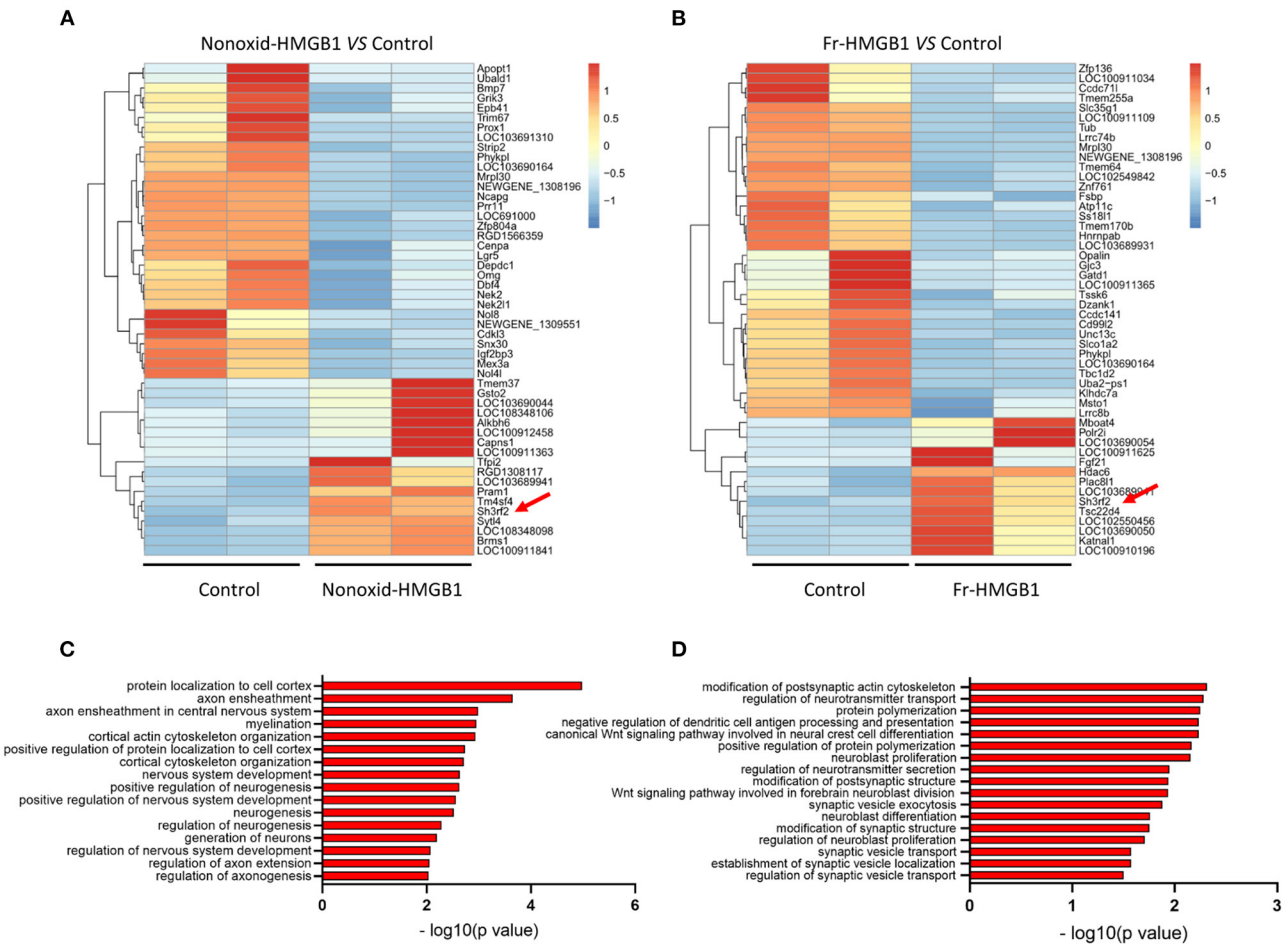
The transcriptome sequence and bioinformatics analysis. **(A)** The heat map of top 50 DEGs in the nonoxid-HMGB1 group vs. the control group. **(B)** The heat map of top 50 DEGs in the fr-HMGB1 group vs. the control group. **(C)** GO analysis for DEGs in the nonoxid-HMGB1 group compared with the control group. **(D)** GO analysis for DEGs in the fr-HMGB1 group compared with the control group.

To identify genes and pathways involved in the promotion of neurite outgrowth and regeneration, we performed RNA-seq analysis. Heatmap showed clear clusters of up- and downregulated genes (Supplementary Figure 2A). Through differential expression analysis of RNA-Seq data, we identified top 50 DEGs (differential expressed genes) (Figures 2A,B). Gene ontology (GO) analysis revealed that the majority of biological processes had correlations with neurodevelopment and nerve regeneration, such as nervous system development, axon extension, neuroblast proliferation, neurotransmitter secretion, myelination, cortical cytoskeleton organization, and so on (Figures 2C,D). In addition, we also found that four accepted pathways for nerve regeneration were activated to varying degrees by nonoxid-HMGB1 (Supplementary Figures 2B–E). As nonoxid-HMGB1 and fr-HMGB1 have the similar activity, they may regulate neurite outgrowth and regeneration by the same mechanism. To identify the potential downstream target of nonoxid-HMGB1 and fr-HMGB1, we compared two sets of DEGs data, and a co-upregulated gene SH3RF2 was detected (Figures 2A,B).

### Expression and Distribution of SH3RF2 in Cortical Neurons

**Figure 3:**
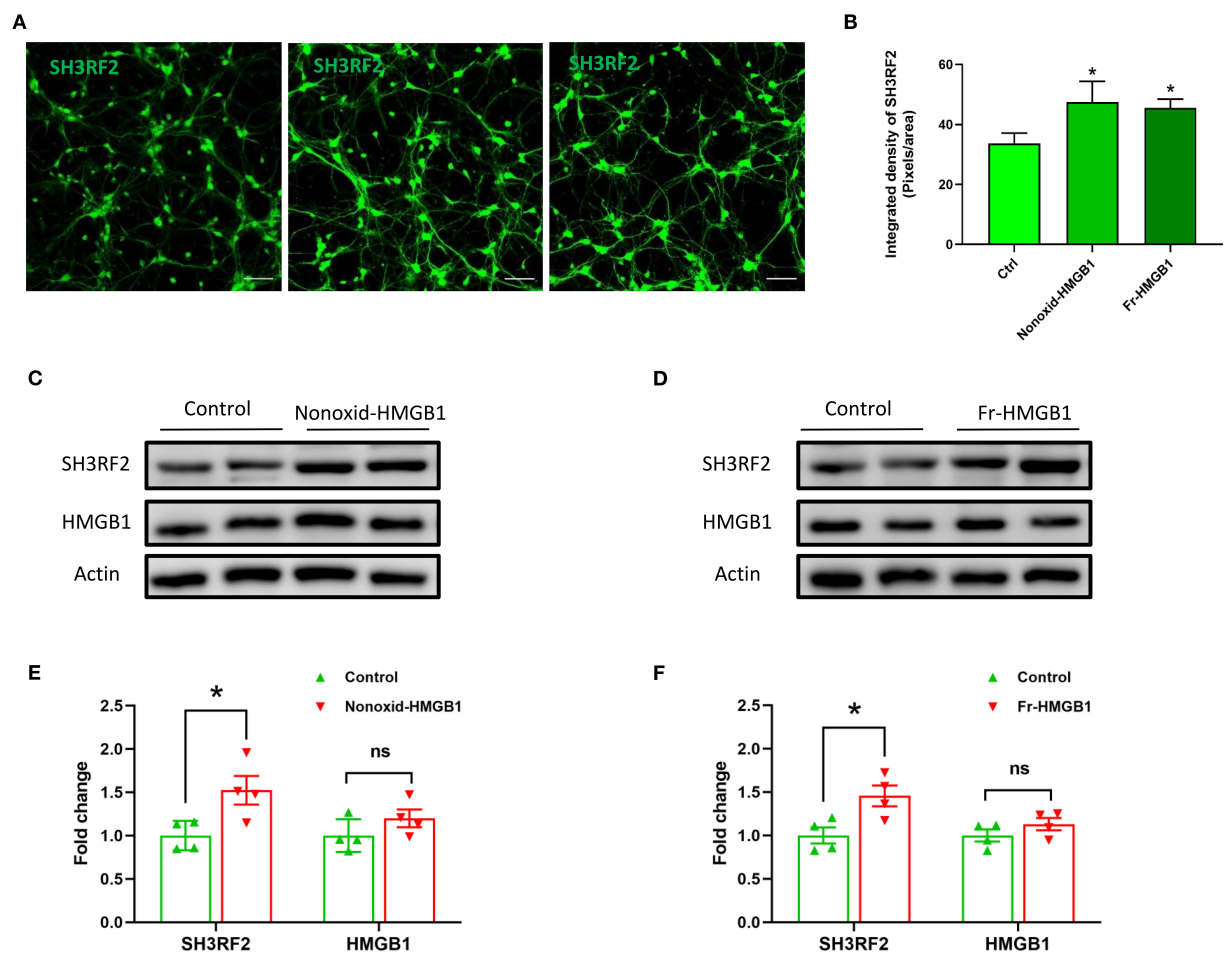
Validation and expression analysis of SH3RF2 in neurons after being treated with nonoxid-HMGB1 or fr-HMGB1. **(A)** Representative fluorescence images of neurons. Scale bars, 50 um. **(B)** Relative quantification of the fluorescence signal. **(C–F)** Western blot for SH3RF2 and HMGB1. Mean ± SEM, *p < 0.05.

To validate the expression of SH3RF2 in neurons, we performed immunostaining of SH3RF2. The results showed that green fluorescence intensity of SH3RF2 was significantly increased in the nonoxid-HMGB1 group and the fr-HMGB1 group as compared with the control (Figures 3A,B). Interestingly, we found that SH3RF2 was widely distributed in the cell bodies and neurites (Figure 3A). To further evaluate the effect of extracellular HMGB1 on the expression of SH3RF2 and HMGB1 in neurons, western blot analysis was performed. As expected, we detected the relative protein level of SH3RF2 was significantly increased in the nonoxid-HMGB1 group and the fr-HMGB1 group. In addition, we found that extracellular nonoxid-HMGB1 and fr-HMGB1 did not influence the expression of HMGB1 in neurons (Figures 3C–F). These results confirmed the expression change of SH3RF2, which were consistent with the bioinformatic analysis result. SH3RF2 could be the potential downstream target of nonoxid-HMGB1 and fr-HMGB1 to participate in neurite outgrowth and axon elongation.

### Nonoxid-HMGB1 Enhances Axon Growth by SH3RF2

**Figure 4:**
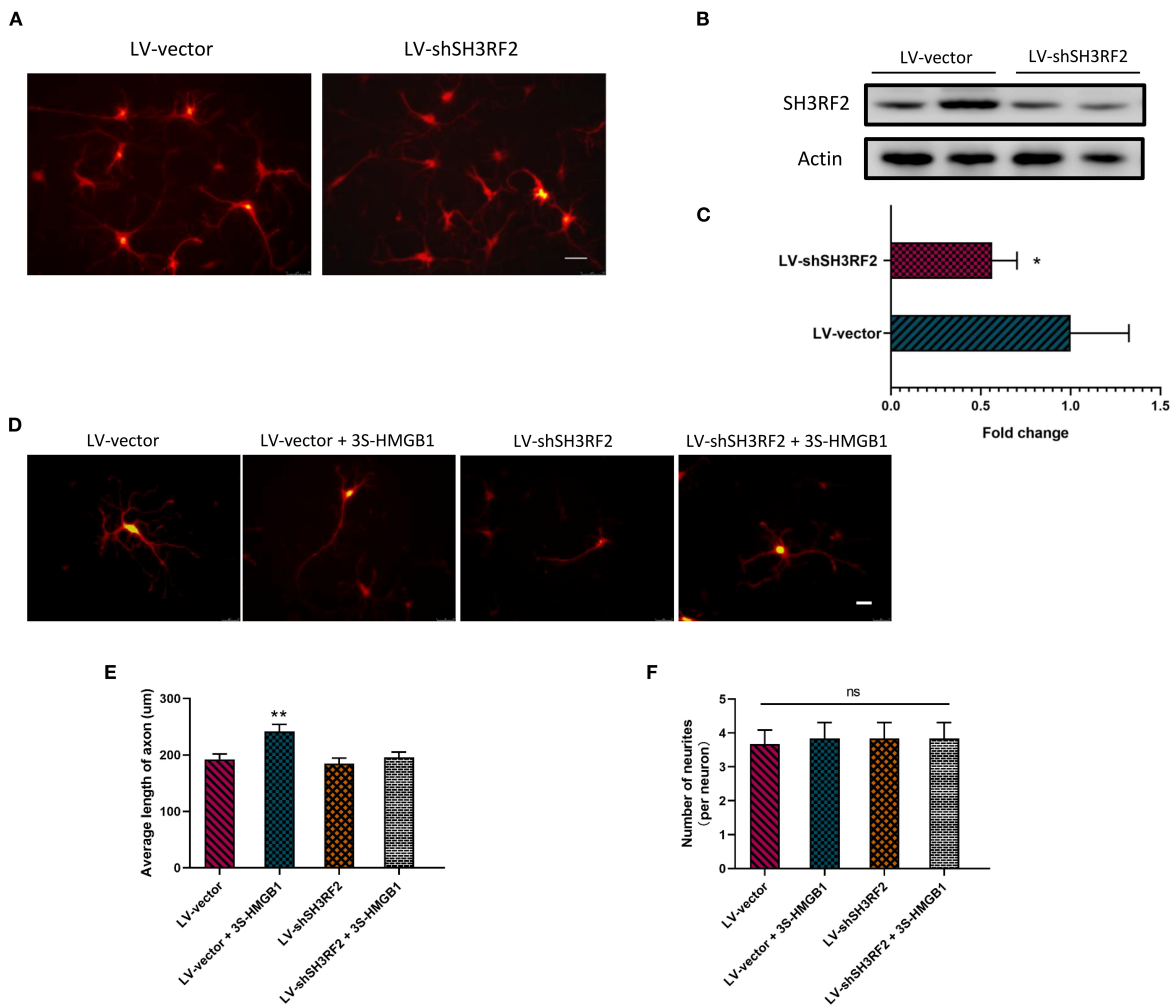
Nonoxid-HMGB1 promotes axon growth by SH3RF2. **(A)** Representative images of neurons after transfection for 72 h. Scale bars, 50 um. **(B,C)** Assessment of silencing efficiency by relative quantification of SH3RF2. **(D,E)** Representative electron microscopy images show that nonoxid-HMGB1 promotes axon growth in control (LV-vector) primary neurons, and it does not cause marked changes of axon growth in shSH3RF2 primary neurons. Scale bars, 50 um. **(F)** Treatment with nonoxid-HMGB1 did not alter the neurites number in control (LV-vector) primary neurons and in shSH3RF2 primary neurons. Mean ± SEM, *p < 0.05, **p < 0.01, ns: no significance.

To validate the potential downstream target gene SH3RF2, we designed this experiment. Because of the instability of fr-HMGB1 in the oxidizing environment, a mutant of fr-HMGB1, nonoxid-HMGB1 (3S-HMGB1), was adopted in this experiment. LV-shSH3RF2 was designed to silence SH3RF2, and empty vectors (LV-vector) were used as control lentivirus. Neurons were transfected with lentivirus at day in Culture 7. After transfection for 72 h, a great number of neurons with strong red fluorescence were observed (Figure 4A), and the SH3RF2 level decreased significantly in the LV-shSH3RF2 group (Figures 4B,C). After infection, nonoxid-HMGB1 was added to the cultured neurons and remained present until fixation after 24 h. Compared with the LV-vector group, the average length of axons was increased in LV-vector + 3S-HMGB1 group. Moreover, no differences were observed between the LV-vector controls and the LV-shSH3RF2 group in axon length. As expected, there was also no diversity between the LV-vector group and the LV-shSH3RF2 + 3S-HMGB1 group (Figures 4D,E). The number of neurites per neuron was no change in different groups (Figure 4F). Taken together, these results demonstrate that silence SH3RF2 antagonizes nonoxid-HMGB1-induced axon growth.

### Nonoxid-HMGB1 Attenuates Object Recognition Memory Deficits in CCI Rats

**Figure 5:**
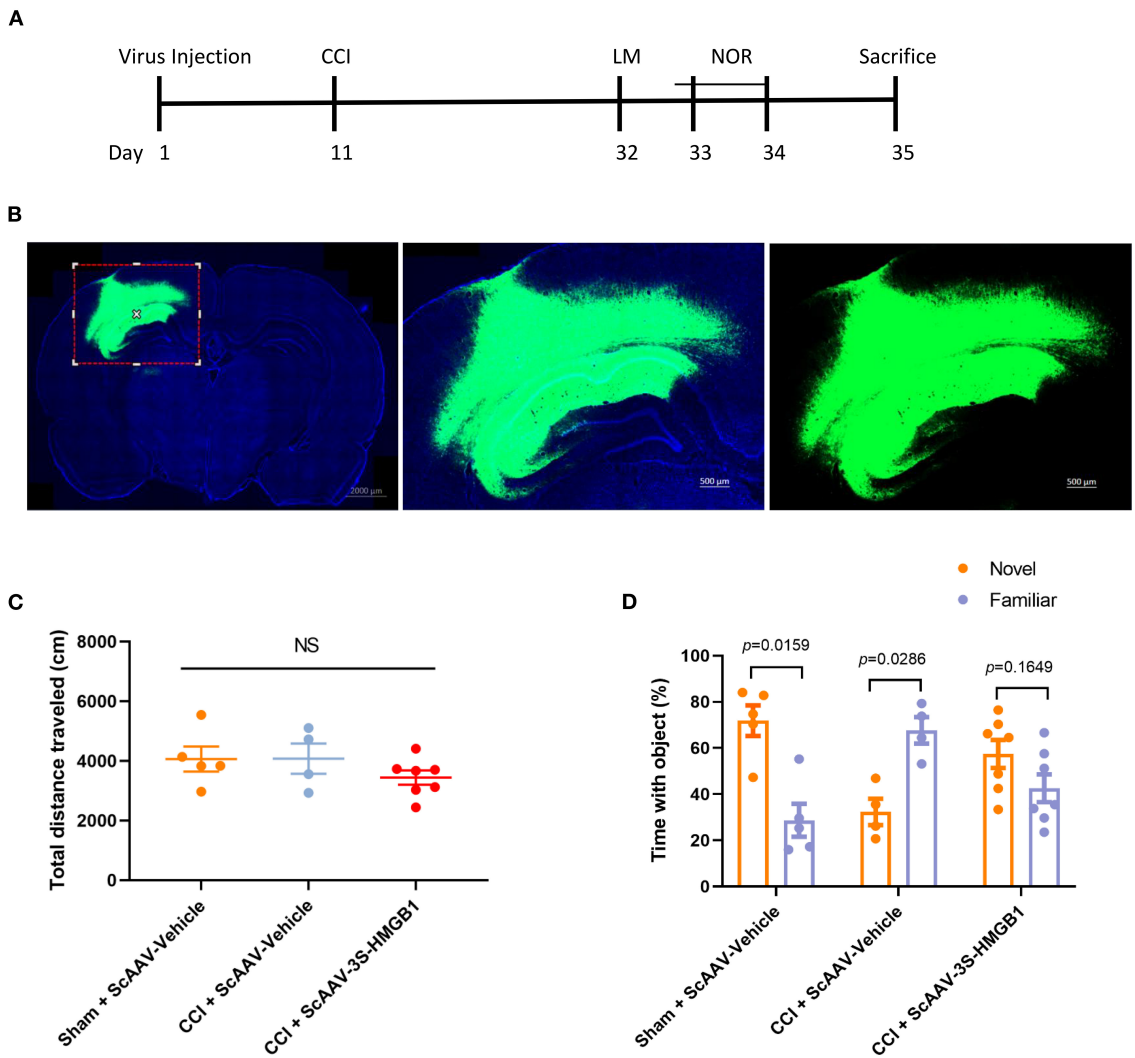
Nonoxid-HMGB1 attenuates learning and memory impairments in CCI rats. **(A)** The experimental timeline for the neurobehavioral testing. **(B)** Representative electron microscopy images of brain sections from rats intervened with ScAAV-3S-HMGB1 or ScAAV-Vehicle for 10 days. **(C)** In the open field test, no differences were observed among three experimental groups. **(D)** Exploration times and discrimination indices were calculated in NOR test. Mean ± SEM; NS: no significance.

To further investigate *in vivo* effects of nonoxid-HMGB1, the CCI model was employed; we injected an ScAAV-3S-HMGB1 expressing vector to increase levels of nonoxid-HMGB1 after TBI, using injections of an empty vehicle as the matched group (ScAAV-Vehicle). The experimental CCI was established at 10 days after ScAAV injection. OF test and novel NOR test were conducted at 3 weeks after CCI (Figure 5A). After virus injection for 10 days, strong green fluorescence was observed in the cortex and hippocampus, and nonoxid-HMGB1 was strongly expressed (Figure 5B, Supplementary Figure 3). We examined the locomotor activity of rats with the OF test, and all three experimental groups displayed similar locomotor activity post-TBI (Figure 5C). For the NOR test, as presented in Figure 5D, the new object reference index in the CCI + ScAAV-Vehicle group was lower than Sham + ScAAV-Vehicle group, indicating that CCI results in an impairment in memory behavior. In the ScAAV-3S-HMGB1 group, the new object preference index was higher than CCI + ScAAV-Vehicle group, but lower than Sham + ScAAV-Vehicle group (Figure 5D). Taken together, these results indicate that nonoxid-HMGB1 improved rats' learning and memory function to a certain extent.

### SH3RF2 Is Required for Nonoxid-HMGB1 Improving CCI-Induced Learning and Memory Damage

**Figure 6:**
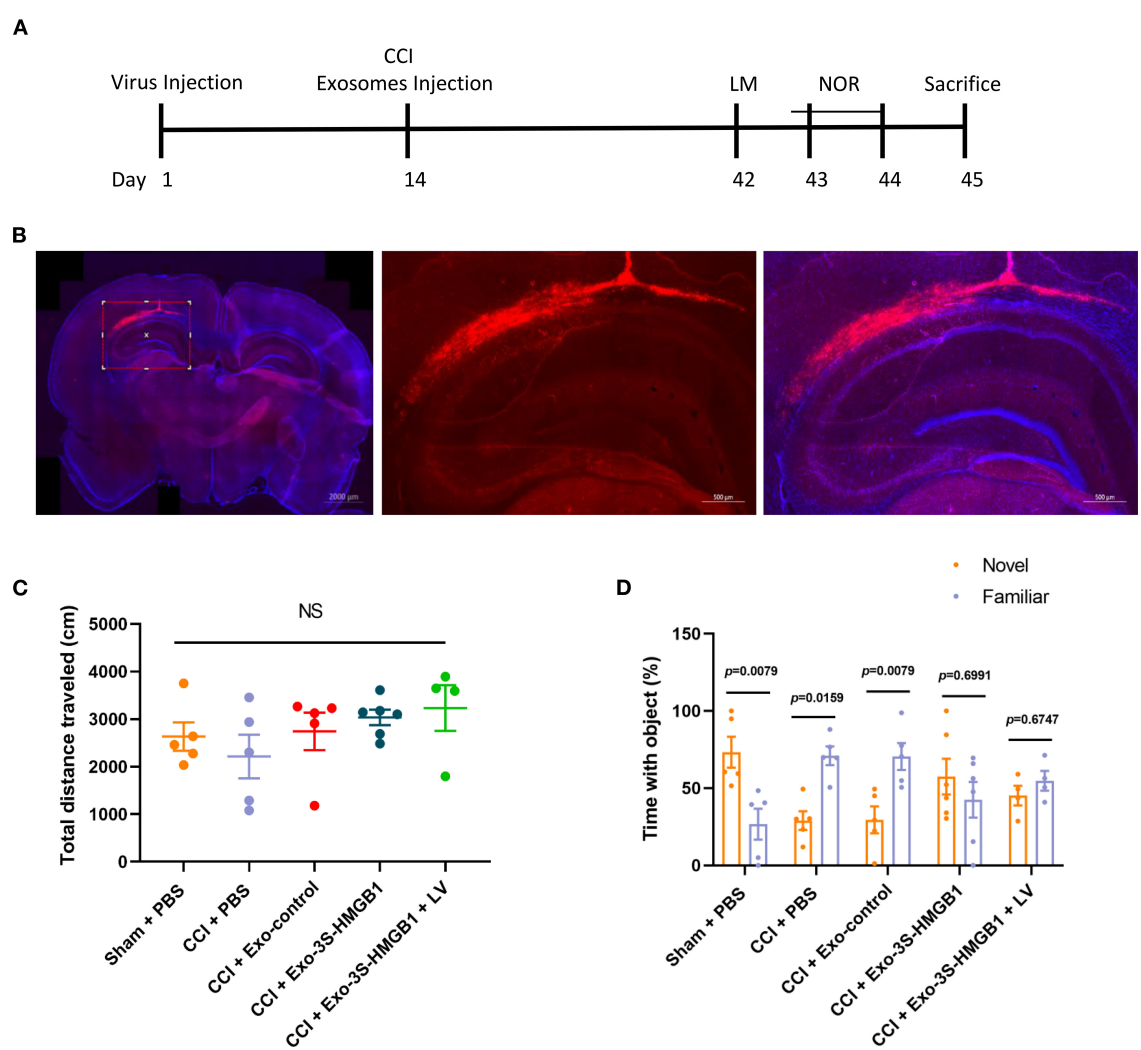
LV-shSH3RF2 attenuated the effect of Exo-3S-HMGB1 to improve the recovery of memory function in CCI. **(A)** The experimental timeline for the neurobehavioral testing. **(B)** Representative electron microscopy images of brain sections from rats intervened with LV-shSH3RF2. **(C)** No differences were observed in the open field test. **(D)** Exploration times and discrimination indices were calculated in NOR test. Mean ± SEM, NS: no significance.

To further disclose whether SH3RF2 is required for nonoxid-HMGB1 improving CCI-induced learning and memory impairments, we knocked down the expression of SH3RF2 in hippocampus by lentivirus (LV-shSH3RF2). Nonoxid-HMGB1-enriched exosomes (Exo-3S-HMGB1) were employed to increase levels of nonoxid-HMGB1 in brain after CCI. The schematic timeline of experiments was presented in Figure 6A.

Exo-3S-HMGB1 was first constructed, and then was administrated into rats after the establishment of CCI. To construct Exo-3S-HMGB1, the BMSCs with high expression of nonoxid-HMGB1 were generated by plasmid transfection, and the green fluorescence was obviously observed in BMSCs after transfection (Supplementary Figures 4A,B). Then, exosomes were identified by analyzing their shape and size (Supplementary Figure 4C) and detecting the specific protein TSG101 and Flotillin-1 (Supplementary Figure 4D). In addition, nonoxid-HMGB1 was detected in exosomes of the transfected BMSCs (Supplementary Figure 4E). All of these results suggest that the Exo-3S-HMGB1 was successfully constructed.

The subsequent study was aimed to explore whether knockdown of SH3RF2 could eliminate the recovery of memory function induced by nonoxid-HMGB1 in CCI. We injected LV-shSH3RF2 to knocked-down SH3RF2 in hippocampus, and decreased SH3RF2 expression was observed after 2 weeks (Figure 6B). Next, we constructed the TBI model of rats and injected exosomes into rats. The OF test results showed that there were no significant differences in total distance traveled among all five groups, suggesting that motor abilities did not interfere with NOR training and test (Figure 6C). For the NOR test, the preference index of a new object in the CCI-PBS group and the CCI-Exo-control group was both significantly lower than a familiar object, indicating that Exo-control could not improve the memory impairment caused by CCI. Rats injected with Exo-3S-HMGB1 showed an improvement in memory function. However, SH3RF2 silencing attenuated the improvement of memory defect induced by Exo-3S-HMGB1 (Figure 6D). All of these data indicated that SH3RF2 silencing could partly eliminate the recovery of memory function induced by nonoxid-HMGB1.

## Discussion

As mentioned before, extracellular HMGB1 exists in three different redox states, and different forms of HMGB1 interact with different receptors (13). Several lines of evidence support that ds-HMGB1 serves as a proinflammatory cytokine *via* interactions with TLR4 (14, 15), whereas fr-HMGB1 mediates tissue regeneration by binding to RAGE (12). Interestingly enough, RAGE signaling has been demonstrated to promote neurite outgrowth and nerve regeneration (31–36). In addition, HMGB1-RAGE axis has been turned out to mediate cell migration and tissue regeneration (37, 38). However, few studies have investigated the potential relationship between different redox states of HMGB1 and nerve growth or regeneration. Against this background, we sought to further confirm if HMGB1 would enhance neurite outgrowth of neurons and, if so, by which kind of redox state. We found that nonoxid-HMGB1 (3S-HMGB1) and fr-HMGB1 promote neurite outgrowth and axon elongation, which is consistent with prior reports that HMGB1 contributes to facilitate neurogenesis and neural regeneration (20–22). However, the neurite outgrowth and axon elongation of neuron were inhibited by ds-HMGB1. Frank et al. reported that ds-HMGB1, but not fr-HMGB1, contributes to inflammatory responses (39). This phenomenon might be caused by activating TLR4 and evoking the production of proinflammatory cytokines subsequently (14, 15).

In order to understand the molecular mechanism through which nonoxid-HMGB1 and/or fr-HMGB1 acts in neurons, we have performed RNA-seq analysis. We noted that nonoxid-HMGB1 shows stronger effects than fr-HMGB1 in genes related to neurogenesis, axon extension, and myelination, but weaker influences on neuroblast proliferation, neuroblast differentiation, and neurotransmitter transport. The divergent results may be due to the inability of nonoxid-HMGB1 to be oxidized to other forms of HMGB1. Notably, we found that SH3RF2 is a co-upregulated DEG in the nonoxid-HMGB1 group and the fr-HMGB1 group. SH3RF2 is a multidomain scaffold protein participated in promoting cell survival, and the knockdown of SH3RF2 promoted apoptosis of cultured cortical neurons (40, 41). Besides, mice with SH3RF2 haploinsufficiency exhibit synaptic plasticity deficits and synaptic dysfunction (42). In this study, we revealed that nonoxid-HMGB1 promotes neurite outgrowth and axon elongation in neurons by increasing SH3RF2 expression. Combined with previous pieces of research, we speculated that SH3RF2 might be a target gene in HMGB1-RAGE axis.

Prior pieces of evidence have indicated that HMGB1 is implicated in neuroinflammation in TBI and exacerbates neurocognitive impairments (17, 18). The release of HMGB1 from damaged tissues has been reported to enhance cerebral edema and neurological deficits (43, 44). In addition, some studies declared that the use of HMGB1 antagonists could reduce cerebral edema, suppress pro-inflammatory cytokine release and microglial activation, and improve neurological outcomes (45–47). As the cysteines of HMGB1 are easily oxidized in oxidizing milieu (13), few studies have further investigated the role of different redox states of HMGB1 in TBI. Interestingly, we found that nonoxid-HMGB1 effectively ameliorates cognitive function, and the inhibition of SH3RF2 attenuated the beneficial effects of nonoxid-HMGB1 on cognitive function. The behavioral improvement induced by nonoxid-HMGB1 post-TBI might result from the neurite outgrowth and axon regeneration of neurons.

Notably, this study has certain limitations. HMGB1 is released from injured cells or death cells (13). Extracellular HMGB1 mainly exists in the form of Disulfide HMGB1 (ds-HMGB1). To avoid these destabilizing factors, we choose a normal cell model in an *in vitro* experiment, but not the neuronal injury model. Further studies are needed to explore the effects of nonoxid-HMGB1 on injured neurons. In addition, the *in vivo* findings indicated that nonoxid-HMGB1 ameliorated cognitive function in rats post-TBI *via* SH3RF2. Further studies will be required to clarify the detailed molecular biological mechanisms in the recovery of cognitive function post-TBI.

Taken together, our findings indicate that nonoxid-HMGB1 reduces TBI-mediated cognitive impairment. This beneficial effect might be through two mechanisms: (i) SH3RF2-induced modulation of neurite outgrowth and regeneration and (ii) SH3RF2-induced modulation of neuronal survival and apoptosis (40, 41). Our experimental results suggest that nonoxid-HMGB1 is an attractive therapeutic candidate for the treatment of TBI, and regulating the redox state of extracellular HMGB1 may be a novel therapeutic approach to treat TBI.

## Data Availability Statement

The raw data supporting the conclusions of this article will be made available by the authors, without undue reservation.

## Ethics Statement

The animal study was reviewed and approved by the Review Committee for the Use of Human or Animal Subjects of Sun Yat-sen University.

## Author Contributions

J-QC performed the experiments and wrote the manuscript. S-QG performed all behavior tests. LL, Z-YJ, and C-FL worked with cell culture and exosome extraction. H-YH and YG designed the project and revised the manuscript. All authors contributed to the article and approved the submitted version.

## Funding

This work was supported by the Science and Technology Program of Guangzhou, China under (Grant No. 201604020080 to YG) and the Guangdong Basic and Applied Basic Research Foundation under (Grant Nos. 2018B0303110014 and 2020B090924004 to YG).

## Conflict of Interest

The authors declare that the research was conducted in the absence of any commercial or financial relationships that could be construed as a potential conflict of interest.

## Publisher's Note

All claims expressed in this article are solely those of the authors and do not necessarily represent those of their affiliated organizations, or those of the publisher, the editors and the reviewers. Any product that may be evaluated in this article, or claim that may be made by its manufacturer, is not guaranteed or endorsed by the publisher.

## Supplementary Material

Supplementary Figure 1 Identification of cultured cortical neurons. After 7 days in culture, two specific markers for neurons were used to identify the cultured cells. The expression of NeuN and MAP2 was detected in the cells.

Supplementary Figure 2 Bioinformatics analysis. **(A)** The heat map illustrates the intensity of signals about transcription for each sample. **(B–E)** Four classical pathways for nerve regeneration were activated to varying degrees by non-oxidized HMGB1.

Supplementary Figure 3 The expression of nonoxid-HMGB1 in neurons. **(A)** Representative images of neurons after ScAAV transfection. **(B)** Western blot for nonoxid-HMGB1.

Supplementary Figure 4 The construction and identification of Exo-3S-HMGB1. **(A)** Representative electron microscopy image of ordinary BMSCs. **(B)** BMSCs transfected with plasmid. Scale bars, 100 um. **(C)** Representative electron microscope image of exosomes. Scale bars, 200 nm. **(D)** Western blot for the markers of exosome TSG101 and Flotin. **(E)** Nonoxid-HMGB1 was detected in the modified exosomes derived from BMSCs.
